# Ultrathin perpendicular magnetic anisotropy CoFeB free layers for highly efficient, high speed writing in spin-transfer-torque magnetic random access memory

**DOI:** 10.1038/s41598-019-54466-7

**Published:** 2019-12-19

**Authors:** Jodi M. Iwata-Harms, Guenole Jan, Santiago Serrano-Guisan, Luc Thomas, Huanlong Liu, Jian Zhu, Yuan-Jen Lee, Son Le, Ru-Ying Tong, Sahil Patel, Vignesh Sundar, Dongna Shen, Yi Yang, Renren He, Jesmin Haq, Zhongjian Teng, Vinh Lam, Paul Liu, Yu-Jen Wang, Tom Zhong, Hideaki Fukuzawa, Po-Kang Wang

**Affiliations:** TDK-Headway Technologies, Inc. 463 S. Milpitas Boulevard, Milpitas, CA 95035 USA

**Keywords:** Spintronics, Materials for devices

## Abstract

Perpendicular magnetic anisotropy (PMA) ferromagnetic CoFeB with dual MgO interfaces is an attractive material system for realizing magnetic memory applications that require highly efficient, high speed current-induced magnetic switching. Using this structure, a sub-nanometer CoFeB layer has the potential to simultaneously exhibit efficient, high speed switching in accordance with the conservation of spin angular momentum, and high thermal stability owing to the enhanced interfacial PMA that arises from the two CoFeB-MgO interfaces. However, the difficulty in attaining PMA in ultrathin CoFeB layers has imposed the use of thicker CoFeB layers which are incompatible with high speed requirements. In this work, we succeeded in depositing a functional CoFeB layer as thin as five monolayers between two MgO interfaces using magnetron sputtering. Remarkably, the insertion of Mg within the CoFeB gave rise to an ultrathin CoFeB layer with large anisotropy, high saturation magnetization, and good annealing stability to temperatures upwards of 400 °C. When combined with a low resistance-area product MgO tunnel barrier, ultrathin CoFeB magnetic tunnel junctions (MTJs) demonstrate switching voltages below 500 mV at speeds as fast as 1 ns in 30 nm devices, thus opening a new realm of high speed and highly efficient nonvolatile memory applications.

## Introduction

PMA-MTJs composed of a CoFeB free layer and MgO tunnel barrier have yielded numerous breakthroughs including scalability, endurance, and high thermal stability^1–9^. From a technological perspective, CoFeB-MgO-based spin-transfer-torque magnetic random access memory (STT-MRAM) has emerged as the most promising alternative to existing silicon-based memory technologies. However, one prominent shortcoming of conventional CoFeB-MgO structures is the requirement for lower switching voltage at faster switching speeds. To attain a low switching voltage, the conservation of spin angular momentum stipulates the need for low magnetic moment which is embodied by an ultrathin ferromagnetic free layer. PMA in ultrathin Fe has been demonstrated^10^, but the inability to achieve PMA in ultrathin CoFeB with dual MgO interfaces stems from the delicate balance between achieving layer continuity, preserving free layer metallicity, and retaining a low resistance-area product (RA) for the MgO layers. Here, we report the use of an Mg insertion layer within an ultrathin CoFeB layer that gives rise to PMA. The device performance of this ultrathin free layer was recently reported as it achieved breakthroughs in low magnetic moment and low damping that gave rise to ultra-low voltage and ultra-low power devices^11,12^. In this work, we show that the achievement of ultrathin CoFeB layers are of technological importance as they fulfill the requirements  for reversal of the free layer magnetization at short pulse lengths below 10 ns, and at deep error rates of less than 1 ppm which are essential to applications such as last level cache that use static random access memory technology.

Conventional free layers use thicker CoFeB (CFB) layers that consist of refractory metal interfaces or insertions^13–15^. These free layers can exhibit good thermal stability withstanding 400 °C back end of line process temperatures and a wide range of operating temperatures, but require a high switching voltage. By contrast, the conservation of spin angular momentum argues that a lower moment free layer is important for the design of low switching voltage magnetic random access memory (MRAM) devices^12,16^. This study shows that ultrathin, thermally stable, high saturation magnetization CFB free layers can be achieved with the incorporation of Mg, enabling a low switching voltage at short pulse lengths. The effect of Mg is remarkable as PMA is observed in CFB free layers with thicknesses *t*_*FL*_ as low as 6.6 Å, whereas in the absence of Mg, PMA is first observed for an 11 Å CFB free layer. For a 30 nm device, estimates of the upper limit of the energy barrier *E*_*b*_ between parallel and anti-parallel states are large, thus good thermal stability is expected up to the maximum operating temperature of 85 °C. Experimentally, nominal 30 nm devices with a 9 Å free layer exhibit sharp switching of the free layer magnetization with respect to applied field or current, and a very low RA of 3.5 Ω·μm^2^ with a tunnel magnetoresistance ratio (TMR) up to 150%. High speed testing revealed these devices are capable of switching at 1 ns with a writing voltage below 500 mV, and below 300 mV for pulse lengths ranging from 3 to 50 ns. These properties highlight the promise of PMA-MTJs, consisting of ultrathin CFB free layers with dual MgO interfaces, for expanding MRAM into new memory applications.

## Results

**Figure 1:**
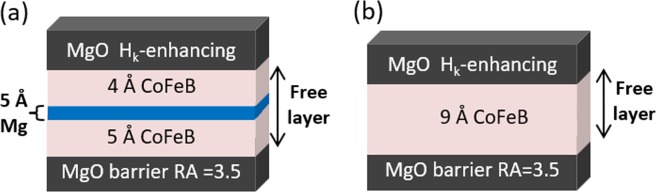
Schematics of ultrathin 9 Å CFB free layers. (**a**) A 9 Å CFB free layer with a 5 Å Mg insertion layer. Layer thicknesses are not drawn to scale. (**b**) A 9 Å CFB free layer without an Mg insertion. Layer thicknesses are not drawn to scale. The MgO barrier of the MTJ stacks was engineered for a low RA of 3.5 Ω·μm^2^.

We report on full MTJ film stacks that include a seed layer, synthetic antiferromagnet reference layer, MgO barrier, CFB free layer, MgO *H*_*k*_-enhancing layer, and nitride-based cap. As depicted in Fig. 1, two variations of the CoFeB free layer are investigated. Each free layer is an ultrathin, 9 Å CoFeB free layer that consists of Fe-rich (CoFe)_1-*y*_B_*y*_, where the Co:Fe ratio is 1:2.7 and *y* is 24 percent. The first free layer variation, shown in Fig. 1a, depicts the insertion of an Mg layer with a nominal thickness of 5 Å within the CFB free layer. The second variation, illustrated in Fig. 1b, omits the Mg layer in favor a continuous CFB layer. All samples are annealed at 400 °C for 3.5 hours after the deposition of the MTJ stack was complete.

**Figure 2:**
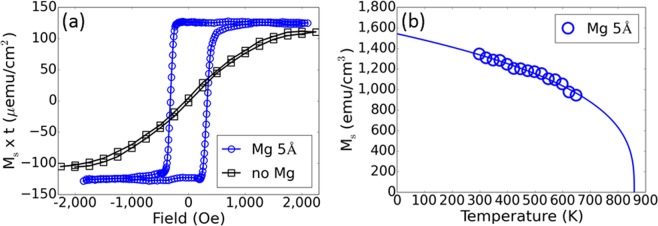
Magnetic properties of ultrathin 9 Å CFB free layers. (**a**) Magnetic moment as a function of applied field for a 9 Å CFB free layer with and without a 5 Å Mg insertion layer. Measurements were performed at room temperature. (**b**) Temperature dependence of *M*_*s*_ for a 9 Å CFB free layer with 5 Å Mg insertion layer.

We use vibration sample magnetometry (VSM) to measure the magnetic properties of the free layer along the out-of-plane easy direction at temperatures between 25 °C and 375 °C. Figure 2a displays hysteresis loops of magnetic moment versus applied magnetic field measured at room temperature on blanket films. In the absence of Mg, a 9 Å CoFeB free layer does not exhibit PMA; whereas, the addition of 5 Å of Mg clearly exhibits free layer PMA. Furthermore, the addition of Mg results in a slightly higher *M*_*s*_ × *t*_*FL*_ than a free layer without Mg consistent with better magnetic properties. Figure 2b shows that the addition of Mg to CFB results in a room temperature saturation magnetization *M*_*s*_ of 1350 emu/cm^3^ with *M*_*s*_ disappearing at 860 K (${T}_{{M}_{s}}=0$). The temperature dependence of the free layer *M*_*s*_ is fitted by the *T*^*1/3*^ power law indicated by the solid line, where ${M}_{s}(T)={M}_{0}\times {(1-\frac{T}{{T}_{{M}_{s}}=0})}^{\frac{1}{3}},$ and is consistent with previous studies of CFB free layers and other ferromagnets^15,17,18^.

**Figure 3:**
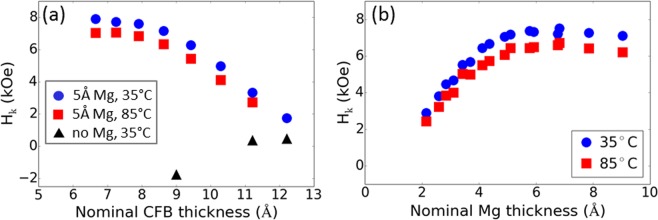
*H*_*k*_ variation with free layer thickness and temperature. (**a**) *H*_*k*_ dependence on nominal CFB thickness measured using FMR at 35 °C and 85 °C, with and without an Mg insertion layer. (**b**) *H*_*k*_ dependence on nominal Mg thickness measured using FMR at 35 °C and 85 °C.

The anisotropy field *H*_*k*_ measured by ferromagnetic resonance spectroscopy (FMR) as a function of the free layer CFB and Mg thickness are depicted with solid symbols in Fig. 3a,b, respectively. In Fig. 3a, free layer PMA and *H*_*k*_ demonstrate a strong dependence on the thickness of CFB and the presence of Mg. Figure 3a reveals a decrease in *H*_*k*_ with CFB thickness due to increasing free layer magnetic moment, *M*_*s*_ × *t*_*FL*_. Interestingly, the addition of Mg to the free layer demonstrates PMA for free layer thicknesses as thin as 6.6 Å and up to 12.2 Å, the lower and upper limits of this study, respectively. In contrast, Fig. 2a displayed the absence of PMA for a no-Mg 9 Å CFB free layer. Without Mg, the triangular symbols in Fig. 3a assert that PMA occurs at an increased CFB thickness of 11 Å with *H*_*k*_ less than 500 Oe at 35 °C. Below this critical thickness, the CFB free layer exhibits in-plane anisotropy. These observations are important as they show that the addition of Mg extends the range of PMA in CFB free layers to ultrathin thickness levels required for low switching voltages in PMA-MTJs.

Next, Fig. 3b demonstrates the sensitivity of the free layer *H*_*k*_ on Mg content. Over the nominal Mg thickness range of this study, 2 Å to 9 Å, a 9 Å CFB free layer demonstrates PMA for all Mg thicknesses. *H*_*k*_ sharply increases from a nominal Mg thickness of 2 Å reaching a maximum near 7 Å. From 7 Å to 9 Å, the *H*_*k*_ dependence on Mg weakened suggesting an upper limit for Mg. This upper limit, and more generally, the range of PMA enabled by the insertion of Mg can be tuned by the oxidation conditions of the adjacent MgO barrier and *H*_*k*_-enhancing layer, nitridation conditions of the nitride-based cap, and Mg content of the often under-oxidized *H*_*k*_-enhancing layer.

**Figure 4:**
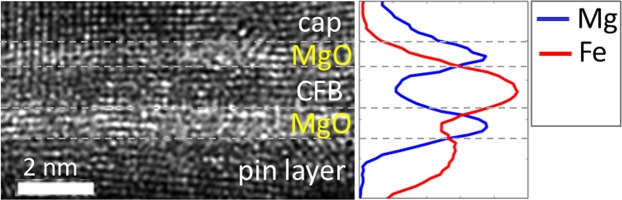
Cross-sectional TEM image of a 9 Å CFB free layer with EDS line profile. TEM and EDS cannot confirm the presence of a distinct Mg layer within the CFB free layer. Dashed lines serve as a guide to the eye.

To better understand the role of the Mg insertion, Fig. 4 shows a cross-sectional transmission electron microscopy (TEM) micrograph and a corresponding energy dispersive spectroscopy (EDS) line scan of a 9 Å CFB free layer with a 5 Å Mg insertion layer. The microstructure of the free layer depicted in the TEM micrograph reveals a continuous, crystalline layer with clear rows of atoms. The ultrathin free layer is believed to benefit from templating effects of the neighboring (001) MgO barrier and *H*_*k*_-enhancing layers, such that the (001) texture appears to extend from the MgO barrier into the nitride-based cap. Surprisingly, both the TEM micrograph and EDS line scan could not confirm the presence of a distinct Mg insertion layer. Therefore, TEM and EDS were extended to an as-deposited sample to understand the effects of annealing on Mg diffusion. Again, unexpectedly, the TEM micrograph and EDS line scan revealed the absence of an Mg layer. Since annealing is not the reason for the absence of the Mg layer, we attribute the absence of the Mg layer to the low binding energy of the Mg-Mg bond (Table 1) that results in the loss of Mg through re-sputtering or the intermixing of Mg during the subsequent deposition of the 4 Å CoFeB layer. We also hypothesize that the 4 Å of CFB may punch through the Mg layer effectively implanting into the lower 5 Å CoFeB layer resulting in a “floating” Mg layer that is partially incorporated into the MgO *H*_*k*_-enhancing layer. Although Mg cannot be accurately quantified in TEM and EDS, a small portion of Mg is believed to remain within the structure. This is supported by the free layer properties which exhibit a strong dependence on Mg content.

**Table 1:** Comparison of bond disassociation energies for constituent free layer atoms.

Bond	Bond Disassociation Energies^22^ (kJ/mol)
Mg-Mg	11.3
Fe-Fe	118
Co-Co	<127

Our experimental results enable us to derive the effective anisotropy constant *K*_*eff*_ and the interfacial anisotropy constant *K*_*i*_ which are defined as: ${K}_{eff}=\frac{{M}_{s}{H}_{k}}{2}=\frac{{K}_{i}}{{t}_{FL}}-2\pi {M}_{s}^{2}$. At 25 °C, a 9 Å CFB free layer with Mg insertion exhibits *M*_*s*_ of 1350 emu/cm^3^ and *H*_*k*_ of 7.3 kOe. These properties correspond to a free layer with *K*_eff_ × *t*_*FL*_ of 0.44 erg/cm^2^ and *K*_*i*_ of 1.48 erg/cm^2^. The anisotropy constants of the 9 Å CFB free layer with Mg are close to values previously reported for thicker free layers designed for high thermal stability and data retention over very wide operating temperature ranges^15^. Given the similarities of the anisotropy constants, MTJs consisting of a 9 Å CFB free layer should exhibit good thermal stability represented by the thermal stability factor Δ. Additionally, with a high ${T}_{{M}_{s}}=0$ and high *H*_*k*_ at 85 °C as shown in Fig. 3, MTJs consisting of a 9 Å CFB free layer should also exhibit good data retention up to 85 °C.

**Figure 5:**
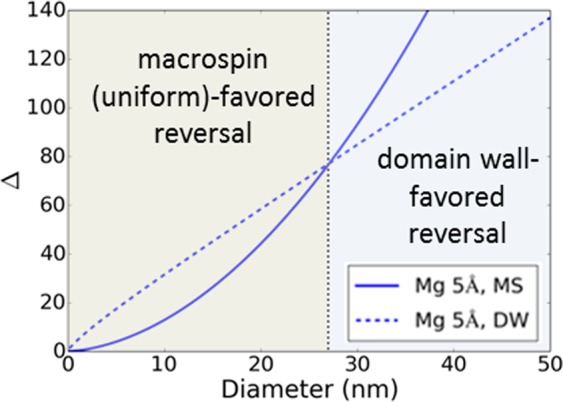
Simulated thermal stability factor Δ for different device diameters under macrospin and domain wall reversal models.

Next, the results of these studies are discussed from the perspective of the requirements for PMA-MTJs for MRAM applications. We can approximate Δ, defined as $\frac{{E}_{b}}{{k}_{B}T},$ using both magnetization reversal models for a circular 30 nm MTJ device fabricated with an ideal, damage-free fabrication process. Under the first model, switching can be described by the macrospin approximation (MS) in which the free layer magnetic moment rotates uniformly. In this case, the energy barrier is given by *E*_*b,MS*_ = ${K}_{eff}S{t}_{FL},$ where *S* is the device surface area^19^. For larger diameters, magnetization reversal is mediated by the nucleation and propagation of a domain wall (DW) across the device leading to the following expression for the energy barrier^20^: *E*_*b,DW*_ = 4 $d{t}_{FL}\sqrt{A\times {K}_{eff}}$, where *d* is the device diameter and *A* is the exchange stiffness constant. The exchange stiffness constant *A* varies as ${M}_{s}^{2\,}$ such that $A={A}_{0,CoFe}{(\frac{{M}_{s,FL}}{{M}_{0,CoFe}})}^{2},$ where ${A}_{0,CoFe}$ = 35.8 × 10^−7^ erg/cm and ${M}_{0,CoFe}$= 1946 emu/cm^3 ^^15,21^. In order to calculate *E*_*b*,_ we must account for the reduction of the demagnetization factor as it relates to *H*_*k*_ using the expressions discussed in ref. ^15^. This relationship finds that *H*_*k*_ for a 30 nm diameter device is 8.9 kOe at 25 °C, and 7.8 kOe at 85 °C. Under a macrospin model of magnetic reversal, a 30 nm device exhibits a high Δ of 93 at 25 °C that decreases to 65 at 85 °C. Under a domain wall model of magnetic reversal, a 30 nm device also exhibits a high Δ of 85 at 25 °C that decreases to 61 at 85 °C. The estimated values of Δ indicate for a 30 nm device, magnetization reversal for a 9 Å CFB free layer occurs by domain wall nucleation and propagation. The crossover for switching mechanisms occurs at a device diameter of 27 nm as shown in Fig. 5. In any case, both reversal models show that ultrathin CFB free layer MTJs are capable of high thermal stability for 30 nm devices between 25 °C and 85 °C. However, as Δ assumes a perfect patterning process in which the cylindrical MTJ pillars incur no damage, the estimated values represent the upper limit for Δ. A previously reported study finds Δ_*MS*_ = 59 at 25 °C and the difference between the estimated Δ and experimental Δ is attributed to MTJ pillar damage incurred during fabrication^12^.

**Figure 6:**
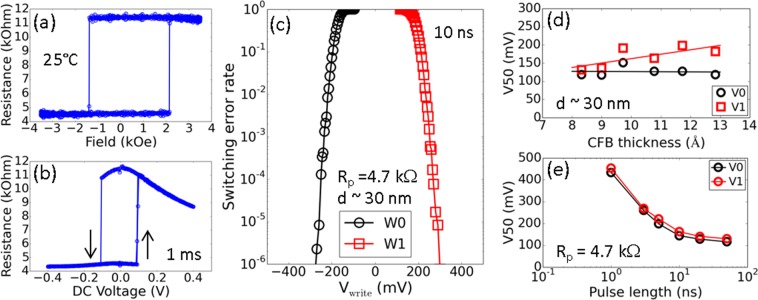
Room temperature device switching characteristics. (**a**) Resistance versus applied magnetic field and (**b**) resistance versus DC voltage (1 ms pulses) curves from a nominal 30 nm device. (**c**) Switching error rate of a nominal 30 nm device measured with 10 ns pulses down to 1 ppm error level. 50% probability switching voltage dependence for nominal 30 nm diameter devices on (**d**) CFB free layer thickness and (**e**) pulse length.

Next, we examine the switching characteristics of nominal 30 nm diameter ultrathin CFB free layer MTJ devices. Figure 6a depicts a resistance versus magnetic field (R-H) loop measured with an applied DC bias of 20 mV. The R-H loop exhibits sharp switching of the free layer between high and low resistance states corresponding to anti-parallel and parallel states, respectively. Figure 6b displays the corresponding resistance versus DC voltage (R-V) loops. R-V measurements consist of sweeping DC voltage between −400 to 400 mV. Again, clear switching of the free layer between anti-parallel and parallel states is observed. The change in resistance is consistent between R-H and R-V loops, and corresponds to a TMR of 150% with a low RA of 3.5 Ω·μm^2^. Note this is not the highest reported TMR for the ultrathin CFB free layer as we have recently reported TMR greater than 180% for a similar structure with a slightly higher RA^12^.

Finally, we discuss the high speed switching characteristics of nominal 30 nm devices summarized in Table 2. Bit error rate tests were first performed with a 10 ns pulse length at an error rate below 10^−6^ or 1 ppm. In Fig. 6c, nominal 30 nm, ultrathin CFB free layer devices demonstrate writing at short pulse lengths with a low switching error rate below 1 ppm. At 1 ppm, the devices exhibit a low writing voltage below 300 mV. The low switching voltage is correlated with the thickness of the CFB free layer as shown in Fig. 6d. While the median 50% switching voltage V0 shows little dependence on the CFB thickness, V1 clearly shows an increase in writing voltage with free layer thickness which is consistent with the conservation of spin angular momentum^12,16^. As an extension of these findings, we look more closely at the ability of these devices to switch at pulse lengths below 10 ns. Figure 6e displays the median 50% switching voltage of nominal 30 nm diameter devices with *R*_*p*_ of 4.7 kΩ as a function of pulse length ranging from 1 ns to 50 ns. We observe switching voltages less than 300 mV for pulse lengths of 3 ns or longer. We also observe switching of the free layer at the fastest switching speed of 1 ns with a writing voltage below 500 mV. These low switching voltages correspond to a low switching energy of 0.056 pJ at 10 ns and 0.044 pJ at 1 ns. These findings highlight the importance of a thin, low moment free layer for the design of efficient, low switching voltage MRAM devices, with the inclusion of Mg serving a critical role for enabling thermally stable, ultrathin PMA CFB free layers.

**Table 2:** Summary of the device properties for an ultrathin 9 Å CFB free layer.

Characteristic	This Work	
Writing pulse length	10 ns	1 ns
Device diameter (electrical CD)	nominal 30 nm	
50% switching voltage, W1	162 mV	453 mV
50% switching current	34 μA	96 μA
Switching energy	0.056 pJ	0.044 pJ
Thermal budget	400 °C for 3.5 hours	
Maximum operating temperature requirement	85 °C	

In conclusion, our studies demonstrate the remarkable role of Mg that enables PMA at ultrathin thicknesses of CoFeB free layers. Without an Mg insertion layer, PMA is first observed for an 11 Å CFB free layer, but with an Mg insertion layer, PMA is observed in CFB free layers as thin as 6.6 Å, the lower limit of this study. A free layer composed of 9 Å CFB with a nominal 5 Å Mg insertion layer, followed by a 3.5 hour annealing at 400 °C, exhibits a high *M*_*s*_ of 1350 emu/cm^3^ and *H*_*k*_ of 7.3 kOe with anisotropy constants identical to conventional, thicker free layers. For 30 nm devices, we observe good thermal stability as the calculated upper limit of Δ is large, approximated at 93 at 25 °C using a macrospin model, and 85 at 25 °C using a domain wall model. At device level, a nominal 30 nm device with a 9 Å free layer exhibits sharp switching of the free layer with a low RA of 3.5 Ω·μm^2^ and TMR of 150%. High speed testing reveal these devices are capable of switching at 1 ns with a writing voltage below 500 mV; and below 300 mV for pulse lengths ranging from 3 to 50 ns. Consistent with the conservation of spin angular momentum, an ultrathin free layer is important for the design of efficient, low switching voltage MRAM devices, and thermally stable, ultrathin  free layers can be achieved with the incorporation of Mg.

## Methods

All MTJ film stacks presented in this work were prepared using magnetron sputtering in an Anelva C-7100 deposition system. After deposition, the blanket film wafers were annealed at 400 °C for 3.5 hours. PMA-MTJ films were also patterned into circular devices with diameters of 30 nm using UV photolithography and etched by a combination of reactive ion etching and argon ion beam etching. Patterned device wafers were annealed at 400 °C for 3.5 hours only after the completion of the fabrication process. Device diameters are electrical diameters calculated from resistance measurements. VSM was used to measure the out-of-plane magnetic moment for temperatures between 25 °C and 375 °C. *M*_*s*_ is defined as the magnetic moment normalized by the nominally-deposited ferromagnetic free layer thickness. FMR was used to measure *H*_*k*_, with error less than 5%, for temperatures between 30 °C and 85 °C. Structural characterization was performed using cross-sectional TEM with elemental compositional analysis performed using EDS. TMR is defined as $\frac{{R}_{AP}-{R}_{P}}{{R}_{P}}.$ The bit error rate is calculated from data collected from up to 1,200,000 test cycles, and defined as the number of write errors divided by the number of write errors and successes for a single device.

## Data Availability

The datasets generated during and/or analysed during the current study are available from the corresponding author on reasonable request.
